# Prevalence, timing and risk factors of extraintestinal manifestations in inflammatory bowel disease: a retrospective cohort study from a German tertiary referral center

**DOI:** 10.1093/crocol/otag100

**Published:** 2026-09-02

**Authors:** Fotios Fousekis, Panagiotis Garantziotis, André Jefremow, Christina Bergmann, Maria Gabriella Raimondo, Andreas Ramming, Georg Schett, Markus F Neurath, Raja Atreya

**Affiliations:** Department of Medicine 1, Uniklinikum Erlangen, Friedrich-Alexander-Universität Erlangen-Nürnberg, Erlangen, Germany; Deutsches Zentrum Immuntherapie, Friedrich-Alexander-Universität Erlangen-Nürnberg, Erlangen, Germany; Deutsches Zentrum Immuntherapie, Friedrich-Alexander-Universität Erlangen-Nürnberg, Erlangen, Germany; Department of Medicine 3 - Rheumatology and Immunology, Uniklinikum Erlangen, Friedrich-Alexander-Universität Erlangen-Nürnberg, Erlangen, Germany; Department of Medicine 1, Uniklinikum Erlangen, Friedrich-Alexander-Universität Erlangen-Nürnberg, Erlangen, Germany; Deutsches Zentrum Immuntherapie, Friedrich-Alexander-Universität Erlangen-Nürnberg, Erlangen, Germany; Deutsches Zentrum Immuntherapie, Friedrich-Alexander-Universität Erlangen-Nürnberg, Erlangen, Germany; Department of Medicine 3 - Rheumatology and Immunology, Uniklinikum Erlangen, Friedrich-Alexander-Universität Erlangen-Nürnberg, Erlangen, Germany; Deutsches Zentrum Immuntherapie, Friedrich-Alexander-Universität Erlangen-Nürnberg, Erlangen, Germany; Department of Medicine 3 - Rheumatology and Immunology, Uniklinikum Erlangen, Friedrich-Alexander-Universität Erlangen-Nürnberg, Erlangen, Germany; Deutsches Zentrum Immuntherapie, Friedrich-Alexander-Universität Erlangen-Nürnberg, Erlangen, Germany; Department of Medicine 3 - Rheumatology and Immunology, Uniklinikum Erlangen, Friedrich-Alexander-Universität Erlangen-Nürnberg, Erlangen, Germany; Deutsches Zentrum Immuntherapie, Friedrich-Alexander-Universität Erlangen-Nürnberg, Erlangen, Germany; Department of Medicine 3 - Rheumatology and Immunology, Uniklinikum Erlangen, Friedrich-Alexander-Universität Erlangen-Nürnberg, Erlangen, Germany; Department of Medicine 1, Uniklinikum Erlangen, Friedrich-Alexander-Universität Erlangen-Nürnberg, Erlangen, Germany; Deutsches Zentrum Immuntherapie, Friedrich-Alexander-Universität Erlangen-Nürnberg, Erlangen, Germany; Department of Medicine 1, Uniklinikum Erlangen, Friedrich-Alexander-Universität Erlangen-Nürnberg, Erlangen, Germany; Deutsches Zentrum Immuntherapie, Friedrich-Alexander-Universität Erlangen-Nürnberg, Erlangen, Germany

**Keywords:** inflammatory bowel disease, extraintestinal manifestations, Crohn’s disease, ulcerative colitis, epidemiology, risk factors, disease timing

## Abstract

**Background:**

Extraintestinal manifestations (EIMs) are a frequent and clinically significant feature of inflammatory bowel disease (IBD), contributing to morbidity and impaired quality of life.

**Objectives:**

To investigate the prevalence, spectrum, timing, and risk factors of EIMs in a large cohort of patients with IBD.

**Methods:**

In this retrospective cohort study, adult patients with ulcerative colitis (UC) or Crohn’s disease (CD) followed at a German tertiary referral center were included. Electronic medical records were reviewed to identify specialist-confirmed EIMs, their timing relative to IBD diagnosis, and associated demographic and disease-related factors.

**Results:**

A total of 817 patients with IBD (527 CD, 290 UC) were included, with a median disease duration of 11 years (IQR 6-19). EIMs were identified in 227 patients (27.8%), with rheumatologic (17.4%) and cutaneous (8.9%) manifestations being the most frequent. Most patients had one EIM (70%), while 30% developed multiple manifestations. Female sex (OR 2.88; 95% CI 2.08-4.02, *P* < .001), longer IBD duration (OR 1.02 per year; 95% CI 1.01-1.04, *P* = .002), and CD (OR 1.57; 95% CI 1.08-2.29, *P* = .018) were independently associated with EIM development. EIMs occurred before or concurrently with IBD diagnosis in 20.7% of affected patients. Kaplan–Meier analysis demonstrated a continuous increase in the cumulative probability of developing EIMs over time, reaching approximately 27% at 15 years of follow-up.

**Conclusion:**

EIMs affect more than one quarter of patients with IBD and may precede or coincide with intestinal disease onset. Female sex and CD appear to be independent risk factors. These findings highlight the importance of early recognition and long-term surveillance of EIMs in patients with IBD.

## Introduction

Ulcerative colitis (UC) and Crohn’s disease (CD) are chronic, immune-mediated conditions with a relapsing and remitting course, representing the two main forms of inflammatory bowel disease (IBD). Although UC and CD primarily affect the gastrointestinal tract, extraintestinal manifestations (EIMs) are frequently observed. Over the course of the disease, the reported prevalence of EIMs varies across studies and may reach up to 47% of patients with IBD.^1–3^ The pathogenesis of EIMs in IBD has not been fully elucidated and seems to be multifactorial. Genetic susceptibility, immune dysregulation, gut barrier dysfunction and microbial factors interact to promote systemic inflammation and contribute to the development of EIMs.^4^ Aberrant immune activation and the translocation of microbial antigens may also trigger immune-mediated damage at extraintestinal sites, potentially via mechanisms such as molecular mimicry and dysregulated cytokine signaling.^5^^,^^6^

The occurrence of EIMs in patients with IBD has been associated with increased morbidity and, in some cases, mortality. The presence of EIMs is often correlated with a more severe disease course, characterized by higher rates of hospitalization, greater need for immunosuppressive therapies and increased likelihood of surgical intervention in both CD and UC.^7^ Certain EIMs, including primary sclerosing cholangitis and thromboembolic events, have been linked to an elevated risk of mortality, whereas others, such as musculoskeletal, ocular, and cutaneous manifestations, substantially contribute to disability and impaired quality of life.^8^ According to the ECCO Guidelines, EIMs comprise a broad spectrum of conditions that can be classified as classical inflammatory manifestations, associated immune-mediated diseases, complications of systemic inflammation, or treatment-related manifestations.^9^ Among the classical EIMs, some manifestations, including peripheral arthritis, erythema nodosum, episcleritis, and oral ulcers, are closely related to intestinal disease activity and often improve with effective control of bowel inflammation. In contrast, EIMs with an independent course include axial arthritis, primary sclerosing cholangitis and uveitis, which do not correlate with IBD activity.^10^ Several factors have been associated with an increased risk of EIMs in IBD, including female sex, Crohn’s disease, more extensive disease, longer disease duration, and markers of more severe disease such as smoking, immunomodulatory or biologic therapy and prior IBD-related surgery.^11^

Despite the identification of these associations, existing evidence is largely derived from heterogeneous cohorts with variable study designs, and the inclusion of additional studies from different populations could contribute to more robust analyses and meaningful comparisons across settings. In particular, only a limited number of studies have examined the chronological relationship between the onset of EIMs and the diagnosis of IBD, showing that EIMs may manifest before, concurrently with, or after the diagnosis of IBD.^12^

In this study, we aimed to identify the epidemiology and spectrum of EIMs that were confirmed by specialists in the respective disease areas, to investigate their timing in relation to IBD diagnosis, and to analyze demographic, clinical, and disease-related factors associated with their occurrence in a large cohort of patients with IBD.

## Methods

### Patients

Adult patients with a confirmed diagnosis of IBD who were followed at IBD outpatient clinics of a tertiary referral center in Germany were included. IBD diagnosis was made based on clinical, laboratory, imaging, endoscopic, and histological findings. Full electronic medical records were retrospectively reviewed to retrieve demographic, clinical, and disease-related information, as well as the presence, type, and timing of EIMs. In addition, a predefined set of common comorbidities considered clinically relevant in both the general population and patients with IBD was recorded from the electronic medical records. For inclusion, patients were required to have at least one year of follow-up at our outpatient clinic, and sufficient available medical records for analysis. Patients were typically followed at regular outpatient visits according to routine clinical practice. Patients with incomplete data or an uncertain diagnosis were excluded.

### Definitions

EIMs were recorded only when the diagnosis had been established following evaluation by the appropriate specialist and was supported by relevant clinical findings and laboratory, histological, and/or imaging investigations. Specifically, rheumatologic manifestations were diagnosed by a rheumatologist, cutaneous manifestations by a dermatologist, ocular manifestations by an ophthalmologist, and hepatobiliary manifestations by a gastroenterologist. Rheumatologic manifestations were defined as the presence of one or more of the following conditions: peripheral arthritis, ankylosing spondylitis, non-radiographic axial spondyloarthritis, enthesitis, and dactylitis. Hepatobiliary manifestations were defined as large-duct primary sclerosing cholangitis, small-duct primary sclerosing cholangitis and portal vein thrombosis. Cutaneous manifestations included erythema nodosum, pyoderma gangrenosum, hidradenitis suppurativa, Sweet’s syndrome, stomatitis aphthosa, leukocytoclastic vasculitis, perioral dermatitis and cheilitis granulomatosa. In addition to the above categories, amyloidosis and Raynaud’s syndrome were included because they have been described in association with IBD in the literature. Both conditions were recorded only when the diagnosis had been confirmed by the appropriate specialist and the manifestation was considered to be related to the underlying IBD. Only EIMs documented in the electronic medical record and confirmed by a specialist were included; patient-reported manifestations without specialist confirmation were excluded. EIMs related to pharmacological treatment, including anti–TNF–induced psoriasis, were excluded from the analysis.

The timing of EIM relative to IBD diagnosis was defined in months before or after the diagnosis of IBD and was based on the date on which the diagnosis was established by the respective specialist, rather than the reported onset of symptoms. A timing of zero was assigned when the EIM diagnosis occurred within ±2 weeks of the IBD diagnosis. The 2-week interval was chosen to account for minor differences in documentation dates when both diagnoses were established during the same diagnostic work-up.

Disease location was classified according to the Montréal classification. In CD, L1 refers to terminal ileal disease, L2 to colonic disease, L3 to ileocolonic disease, and L4 to isolated upper gastrointestinal involvement. In UC, E1 refers to proctitis, E2 to left-sided colitis, and E3 to pancolitis.

Concomitant immune-mediated disease was defined as the presence of at least one immune-mediated disorder that was not considered attributable to IBD and was not included among the EIMs evaluated in the present study. These disorders could have been diagnosed either before or after the diagnosis of IBD. Accordingly, no condition was classified both as an EIM and as a concomitant immune-mediated disease.

### Outcomes

The primary outcomes were the prevalence of EIMs and the distribution and frequency of EIMs across categories and subtypes in the overall cohort.

Secondary outcomes included comparisons of EIM prevalence according to sex and IBD subtype (UC vs CD), the number of EIMs per patient (1, 2, or ≥3), and the timing of EIM onset in relation to the diagnosis of IBD (before, concurrently with, or after diagnosis). In addition, demographic and disease-related factors associated with the presence of EIMs were assessed using univariable and multivariable analyses, and interactions between different EIM categories were explored.

### Statistical analysis

Continuous variables were assessed for normality using visual inspection of histograms and the Shapiro–Wilk test and are presented as median with interquartile range (IQR), as most variables showed a non-normal distribution. Categorical variables are reported as absolute numbers and percentages. Comparisons between groups were performed using the Mann–Whitney *U* test for continuous variables and the chi-squared test or Fisher’s exact test, as appropriate, for categorical variables. To account for multiple comparisons in the exploratory univariable analyses presented in Tables 1 and 2, *P*-values were additionally adjusted using the Benjamini–Hochberg false discovery rate (FDR) procedure. To identify factors associated with the presence of EIMs, univariable logistic regression analyses were initially performed. Variables considered clinically relevant or showing an association with EIMs in univariable analyses were subsequently included in a multivariable logistic regression model. Results are reported as odds ratios (ORs) with corresponding 95% confidence intervals (CIs).

**Table 1:** Comparison of patients with and without EIM.

	Overall, *n*: 817	EIM (+), *n*: 227	EIM (-), *n*: 590	*P*-value	FDR-adjusted *P*-value*
**Sex (Female), *n* (%)**	402 (49%)	152 (67%)	250 (42%)	**<.001**	**<.001**
**Age at diagnosis of IBD**	27 (20-38)	27 (19-37)	26 (19-38)	.8	.9
**Median (IQR)**					
**Duration of IBD**	11 (6-19)	13 (7-23)	11 (5-18)	**<.001**	**.005**
**Median (IQR)**					
**Type of IBD [Crohn Disease, *n* (%)]**	527 (65%)	165 (73%)	362 (61%)	**.003**	**.021**
**Smoking (exposure), *n* (%)**	159 (19%)	57 (25%)	102 (17%)	**.015**	.06
**Montréal UC**					
** E1**	20 (6.9%)	4 (6.5%)	16 (7.0%)	>.9	>.9
** E2**	113 (39%)	18 (29%)	95 (42%)	.1	.2
** E3**	157 (54%)	40 (63%)	117 (51%)	.088	.2
**Montréal classification^a^**					
** L1**	127 (24%)	29 (17.6%)	98 (27.1%)	**.024**	.081
** L2**	69 (13%)	23 (14%)	46 (13%)	.8	.9
** L3**	331 (63%)	113 (68%)	218 (60%)	.085	.2
** L4+**	135 (26%)	52 (32%)	83 (23%)	**.047**	.13
** B1**	182 (35%)	58 (35%)	124 (34%)	>.9	>.9
** B2**	235 (45%)	77 (47%)	158 (44%)	.6	.7
** B3**	166 (31%)	47 (28%)	119 (33%)	.4	.5
** Perianal disease**	122 (23%)	50 (30%)	72 (20%)	**.012**	.06
**IBD-related surgery, *n* (%)**	254 (31%)	82 (36%)	172 (29%)	.065	.2
**Family history, *n* (%)**	29 (3.5%)	12 (5.3%)	17 (2.9%)	.15	.2
**History of appendectomy prior to IBD diagnosis, *n* (%)**	44 (5.4%)	15 (6.6%)	29 (4.9%)	.4	.6
**History of tonsillectomy prior to IBD diagnosis, *n* (%)**	24 (2.9%)	10 (4.4%)	14 (2.4%)	.2	.3
**Concomitant immune-mediated disease, *n* (%)**	71 (8.7%)	26 (11.5%)	45 (7.6%)	.1	.2

a Montréal classification percentages are calculated using UC patients as the denominator for E1-E3 and CD patients as the denominator for L1-L4 and B1-B3.

* *P*-values were adjusted for multiple comparisons using the Benjamini–Hochberg false discovery rate (FDR) procedure.

**Table 2:** Stratification and comparison of extraintestinal manifestations by sex and IBD subtype.

Extra-intestinal manifestations	Female (*n*: 152)	Male (*n*: 75)	*P*-value	FDR-adjusted *P*-value	CD (*n*: 165)	UC (*n*: 62)	*P*-value	FDR-adjusted *P*-value
**Number of EIMs**			.4	>.9			.9	>.9
** 1 EIM**	102 (67.1%)	57 (76%)			116 (70.3%)	43 (69.4%)		
** 2 EIM**	43 (28.3%)	15 (20%)			41 (24.8%)	17 (27.4%)		
** ≥3 EIMs**	7 (4.6%)	3 (4.0%)			8 (4.8%)	2 (3.2%)		
**Cutaneous manifestations**								
** Erythema nodosum**	27 (17.8%)	3 (4.0%)	**.004**	.08	21 (12.7%)	9 (14.5%)	.7	>.9
** Pyoderma gangrenosum**	9 (5.9%)	6 (8.0%)	.6	>.9	10 (6.1%)	5 (8.1%)	.6	>.9
** Hidradenitis suppurativa**	10 (6.6%)	2 (2.7%)	.3	>.9	9 (5.5%)	3 (4.8%)	>.9	>.9
** Sweet’s syndrome**	1 (0.7%)	0 (0.0%)	>.9	>.9	1 (0.6%)	0 (0%)	.065	>.9
** Stomatitis aphthosa**	7 (4.6%)	3 (4.0%)	>.9	>.9	10 (6.1%)	0 (0%)	.5	>.9
** Leukocytoclastic vasculitis**	1 (0.7%)	1 (1.3%)	.55	>.9	1 (0.6%)	1 (1.6%)	.5	>.9
** Perioral dermatitis**	1 (0.7%)	0 (0%)	>.9	>.9	1 (0.6%)	0 (0%)	>.9	>.9
** Cheilitis granulomatosa**	0 (0%)	4 (5.3%)	.3	>.9	4 (2.4%)	0 (0%)	.6	.7
**Rheumatologic manifestations**								
** Peripheral arthritis**	71 (46.7%)	31 (41.3%)	.4	>.9	72 (43.6%)	30 (48.4%)	.5	>.9
** Ankylosing spondylitis**	32 (21.1%)	20 (26.7%)	.3	>.9	40 (24.2%)	12 (19.4%)	.4	>.9
** Non-radiographic axial spondyloarthritis**	6 (3.9%)	4 (5.3%)	.7	>.9	9 (5.5%)	1 (1.6%)	.3	>.9
** Enthesitis**	5 (3.3%)	6 (8.0%)	.2	>.9	8 (4.8%)	3 (4.8%)	>.9	>.9
** Dactylitis**	2 (1.3%)	1 (1.3%)	>.9	>.9	1 (0.6%)	2 (3.2%)	.2	>.9
**Hepatobiliary manifestations**								
** Large duct PSC**	11 (7.2%)	8 (10.7%)	.4	>.9	11 (6.7%)	8 (12.9%)	.13	>.9
** Small duct PSC**	1 (0.7%)	0 (0%)	>.9	>.9	1 (0.6%)	0 (0%)	>.9	>.9
** Portal vein thrombosis**	3 (2.0%)	0 (0%)	.6	>.9	3 (1.8%)	0 (0%)	.6	>.9
**Ocular manifestations**								
** Episcleritis**	5 (3.3%)	1 (1.3%)	.7	>.9	4 (2.4%)	2 (3.2%)	.7	>.9
** Scleritis**	2 (1.3%)	0 (0%)	>.9	>.9	0 (0%)	2 (3.2%)	.07	.7
** Uveitis**	12 (7.9%)	3 (4.0%)	.4	>.9	11 (6.7%)	4 (6.5%)	>.9	>.9
**Amyloidosis**	0 (0%)	1 (1.3%)	.3	>.9	1 (0.6%)	0 (0%)	>.9	>.9
**Raynaud syndrome**	4 (2.6%)	1 (1.3%)	>.9	>.9	5 (3.0%)	0 (0%)	.3	>.9

In addition, a multivariable Cox proportional hazards regression analysis was performed to evaluate factors associated with the time to first EIM after IBD diagnosis. Time at risk was calculated from the date of IBD diagnosis to the first EIM or last follow-up. Patients whose first EIM occurred before the diagnosis of IBD were excluded from this analysis because the event preceded the defined time origin, resulting in negative event times. Results are presented as hazard ratios (HRs) with corresponding 95% CIs.

An exploratory interaction analysis between different EIM categories was conducted using pairwise chi-squared tests, and the strength of association was quantified using Cramer’s V statistic. Cramer’s V values were interpreted according to conventional thresholds, with values around 0.10, 0.30, and 0.50 indicating weak, moderate, and strong associations, respectively.

The timing of the first EIM in relation to the diagnosis of IBD was analyzed descriptively and is presented as months before or after IBD diagnosis. Kaplan–Meier survival curves were generated, with the occurrence of the first EIM defined as the event of interest and time calculated from the date of IBD diagnosis to the date of first EIM diagnosis or last follow-up. Patients whose first EIM had been diagnosed before the diagnosis of IBD were considered to have experienced the event at time zero in the Kaplan–Meier analysis. Patients who did not develop EIMs during follow-up were censored at their last recorded visit. Statistical analyses were conducted using R (version 4.5.0; R Foundation for Statistical Computing, Vienna, Austria).

### Ethics

The study was approved by the local institutional ethics committee (Approval No.: 01.04.2026/26-71-Br). The study adhered to the Strengthening the Reporting of Observational Studies in Epidemiology (STROBE) guidelines.^13^

## Results

### Patient characteristics

The study cohort comprised a total of 817 patients with IBD, of whom 290 (35.5%) were diagnosed with UC and 527 (64.5%) with CD (Table 3). The sex distribution was balanced, with 402 females (49.2%) and 415 males (50.8%), and was comparable between UC and CD subgroups. The median age at IBD diagnosis in the overall cohort was 27 years (IQR 20-38), with patients with UC being diagnosed at a slightly older median age compared to those with CD (29 vs 25 years). The median disease duration was 11 years (IQR 6-19), and was longer in CD than in UC (13 vs 9 years). A family history of IBD was reported in 3.5% of patients. Smoking exposure (current or former) was documented in 19% of the total cohort and was more frequent among patients with CD than UC. According to the Montréal classification, the majority of UC patients had extensive disease (E3, 54.1%), while ileocolonic involvement (L3, 63%) and strictures (B2, 45%) predominated in CD. IBD-related surgery had been performed in 31% of patients, with a markedly higher proportion in CD compared to UC (43% vs 9.3%). Prior appendectomy and tonsillectomy were uncommon. A range of comorbidities was observed, including metabolic-associated fatty liver disease, cholelithiasis, thromboembolic events, osteoporosis and depression. 8.7% of patients suffered from another immune-mediated disease in addition to IBD. Regarding treatment exposure, the majority of patients had received biologic therapy, most commonly anti-TNF agents, followed by integrin and interleukin-23 inhibitors. Conventional therapies such as 5-aminosalicylates, systemic corticosteroids and immunomodulators were also frequently used.

**Table 3:** Demographics and clinical characteristics of the patient cohort.

	Total	Ulcerative colitis	Crohn’s disease
**Patients**	817	290 (35.5%)	527 (64.5%)
**Sex**			
**Female**	402 (49.2%)	142 (49%)	260 (49.3%)
**Male**	415 (50.8%)	148 (51%)	267 (50.7%)
**Median age of patient at diagnosis (years) [IQR]**	27 (20-38)	29 (22-42)	25 (19-35)
**Median IBD duration (years) [IQR]**	11 (6-19)	9 (5-16)	13 (6-21)
**Family history of IBD (*n*) [%]**	29 (3.5%)	10 (3.4%)	19 (3.5%)
**Smoking status (exposure) [%]**	159 (19%)	29 (10%)	130 (25%)
**Disease phenotype (Montréal classification)**			
** E1**		20 (6.9%)	
** E2**		113 (39%)	
** E3**		157 (54.1%)	
** L1**			127 (24%)
** L2**			69 (13%)
** L3**			331 (63%)
** L4+**			135 (26%)
** B1**			182 (35%)
** B2**			235 (45%)
** B3**			166 (31%)
** *P***			122 (23%)
**IBD-related surgery (%)**	254 (31%)	27 (9.3%)	227 (43%)
**History of appendectomy prior IBD diagnosis (%)**	44 (5.4%)	12 (4.1%)	32 (6.1%)
**History of tonsillectomy prior IBD diagnosis (%)**	24 (2.9%)	7 (2.4%)	17 (3.2%)
**Concomitant diseases/Comorbidities**			
** Pulmonary embolism**	5 (0.6%)	3 (1.0%)	2 (0.4%)
** Cholelithiasis**	72 (8.8%)	14 (4.8%)	58 (11%)
** Metabolic associated liver disease (MAFLD)**	95 (12%)	26 (9.0%)	69 (13%)
** Gallstones related pancreatitis**	3 (0.4%)	2 (0.7%)	1 (0.2%)
** Drug-induced pancreatitis**	26 (3.2%)	13 (4.5%)	13 (2.5%)
** Other etiology pancreatitis**	14 (1.7%)	6 (2.1%)	8 (1.5%)
** Nephrolithiasis**	29 (3.5 %)	3 (1.0%)	26 (4.9%)
** Osteopenia**	21 (2.6%)	8 (2.8%)	13 (2.5%)
** Osteoporosis**	42 (5.1%)	8 (2.8%)	34 (6.5%)
** Diabetes mellitus**	32 (3.9%)	14 (4.8%)	18 (3.4%)
** Coronary heart disease**	26 (3.2%)	11 (3.8%)	15 (2.8%)
** Deep vein thrombosis**	32 (3.9%)	13 (4.5%)	19 (3.6%)
** Budd-Chiari**	1 (0.3%)	1 (0.3%)	0 (0%)
** Stroke**	8 (1.0%)	3 (1.0%)	5 (0.9%)
** Multiple sclerosis**	5 (0.6%)	1 (0.3%)	4 (0.8%)
** Dementia**	1 (0.1%)	0 (0%)	1 (0.2%)
** Depression**	37 (4.5%)	8 (2.8%)	29 (5.5%)
** Concomitant immune-mediated disease**	71 (8.7%)	21 (7.2%)	50 (9.5%)
**Medication (ever)**			
** 5-ASA**	330 (40%)	188 (65%)	142 (27%)
** Steroid systemics**	385 (47%)	148 (51%)	237 (45%)
** Budesonide**	160 (20%)	54 (19%)	106 (20%)
** Azathioprine/6-MP**	329 (40%)	89 (31%)	240 (46%)
** Methotrexate**	57 (7.0%)	8 (2.8%)	49 (9.3%)
** Infliximab**	556 (68%)	194 (67%)	362 (69%)
** Adalimumab**	251 (31%)	60 (21%)	191 (36%)
** Golimumab**	22 (2.7%)	18 (6.2%)	4 (0.8%)
** Certolizumab**	1 (0.1%)	1 (0.3%)	0 (0%)
** Vedolizumab**	202 (25%)	127 (44%)	75 (14%)
** Ustekinumab**	297 (36%)	76 (26%)	221 (42%)
** Risankizumab**	155 (19%)	21 (7.2%)	134 (25%)
** Mirikizumab**	15 (1.8%)	15 (5.2%)	0 (0%)
** Guselkumab**	17 (2.1%)	10 (3.4%)	7 (1.3%)
** Tofacitinib**	13 (1.6%)	12 (4.1%)	1 (0.2%)
** Upadacitinib**	46 (5.6%)	19 (6.6%)	27 (5.1%)
** Ozanimod**	2 (0.3%)	2 (0.8%)	0 (0%)
** Filgotinib**	10 (1.2%)	7 (2.4%)	3 (0.6%)
** Cyclosporin**	33 (4.0%)	28 (9.7%)	5 (0.9%)

### Types and prevalence of extraintestinal manifestations

EIMs were identified in 227 patients, corresponding to 27.8% of this cohort, with a total of 306 EIMs recorded (Table 4). Among patients with EIMs, the majority presented with a single manifestation (70%), while 25.6% had two EIMs and 4.4% experienced three or more. Rheumatologic manifestations constituted the most frequent EIM category, with 142 (17.4%) patients having at least one rheumatologic manifestation. Peripheral arthritis was the predominant rheumatologic manifestation, followed by ankylosing spondylitis, non-radiographic axial spondyloarthritis, enthesitis and dactylitis. Cutaneous manifestations were the second most common category, observed in 73 patients (8.9% of the cohort). Among these, erythema nodosum and pyoderma gangrenosum were the most frequently reported, while other dermatologic manifestations, including hidradenitis suppurativa and stomatitis aphthosa, were less common. Hepatobiliary manifestations were identified in 23 patients (2.8%), with large-duct primary sclerosing cholangitis representing the majority of cases. Ocular manifestations were observed in a smaller proportion of patients, including episcleritis, scleritis, and uveitis, cumulatively affecting 23 patients. Amyloidosis and Raynaud syndrome were infrequently documented.

**Table 4:** Spectrum and prevalence of extraintestinal manifestations.

Extraintestinal manifestations	Total cohort, *n*: 817 (%)	EIMs cohort *n*: 227 (%)
**Number of EIMs**		
** 1 EIM**	159 (19.5%)	70%
** 2 EIM**	58 (7.1%)	25.6%
** ≥3 EIMs**	10 (1.2%)	4.4%
**Cutaneous manifestations**		
** Erythema nodosum**	30 (3.7%)	13.2%
** Pyoderma gangrenosum**	15 (1.8%)	6.6%
** Hidradenitis suppurativa**	12 (1.5%)	5.3%
** Sweet’s syndrome**	1 (0.1%)	0.5%
** Stomatitis aphthosa**	10 (1.2%)	4.4%
** Leukocytoclastic vasculitis**	2 (0.2%)	0.9%
** Perioral dermatitis**	1 (0.1%)	0.5%
** Cheilitis granulomatosa**	4 (0.5%)	1.8%
**Rheumatologic manifestations**		
** Peripheral arthritis**	102 (12.5%)	44.9%
** Ankylosing spondylitis**	52 (6.4%)	22.9%
** Non-radiographic axial spondyloarthritis**	10 (1.2%)	4.4%
** Enthesitis**	11 (1.3%)	4.8%
** Dactylitis**	3 (0.4%)	1.4%
**Hepatobiliary manifestations**		
** Large duct PSC**	19 (2.3%)	8.6%
** Small duct PSC**	1 (0.1%)	0.4%
** Portal vein thrombosis**	3 (0.4%)	1.3%
**Ocular manifestations**		
** Episcleritis**	7 (0.9%)	3.1%
** Scleritis**	2 (0.2%)	0.9%
** Uveitis**	15 (1.8%)	6.6%
**Amyloidosis**	1 (0.1%)	0.4%
**Raynaud syndrome**	5 (0.6%)	2.2%

Patients may have had more than one manifestation within the same EIM category; therefore, the sum of subtype counts may exceed the number of affected patients.

### Comparison between patients with and without extraintestinal manifestations

Patients with EIMs (*n* = 227) differed significantly from those without EIMs (*n* = 590) in several demographic and disease-related characteristics (Table 1). Female sex was markedly more frequent in the EIM-positive group compared with the EIM-negative group (67% vs 42.4%, FDR-adjusted *P* < .001). Age at IBD diagnosis did not differ between groups. In contrast, disease duration was longer among patients with EIMs (median 13 years [IQR 7-23] vs 11 years [IQR 5-18]; FDR-adjusted *P* = .005). CD was more prevalent in the EIM-positive group (165/227, 72.7%) than in the EIM-negative group (61.4%; FDR-adjusted *P* = .021). Although smoking exposure, ileal disease (L1), upper gastrointestinal involvement (L4+), and perianal disease showed nominal associations in the unadjusted analyses, these associations were no longer statistically significant after FDR adjustment.

Among patients with UC, disease extent according to the Montréal classification did not differ between groups. Likewise, among patients with CD, no significant differences were observed in disease location, disease behavior, or perianal disease after FDR adjustment. IBD-related surgery showed a numerical but not statistically significant difference (36% vs 29%). No significant associations were observed for family history of IBD, appendectomy prior to IBD diagnosis, tonsillectomy prior to IBD diagnosis, or concomitant immune-mediated disease.

### Stratified analysis of extraintestinal manifestations by sex and IBD type

A stratified analysis of EIMs by sex and IBD subtype was performed (Table 2). The distribution of the number of EIMs per patient did not differ significantly between CD and UC. Across almost all cutaneous, rheumatologic, hepatobiliary and ocular manifestations, no statistically significant differences were observed between males and females. Although erythema nodosum showed a nominal association with female sex in the unadjusted analysis (*P* = .004), this association did not remain statistically significant after FDR adjustment (FDR-adjusted *P* = .08).

### Interaction between extraintestinal manifestations and risk factors of extraintestinal manifestations development

An exploratory interaction analysis of EIMs was performed using Cramer’s V to assess the strength of pairwise associations (Figure 1; Table S1). Overall, most associations were weak, indicating limited co-occurrence among the majority of EIMs. The strongest correlation was observed between peripheral arthritis and ankylosing spondylitis (Cramer’s V ≈ 0.20), suggesting clustering within the rheumatologic spectrum. A weak association was also identified between ankylosing spondylitis and ocular manifestations (Cramer’s V ≈ 0.17). In contrast, associations among cutaneous manifestations, including erythema nodosum and pyoderma gangrenosum, were weak. Hepatobiliary manifestations showed minimal association with other EIM categories.

**Figure 1:**
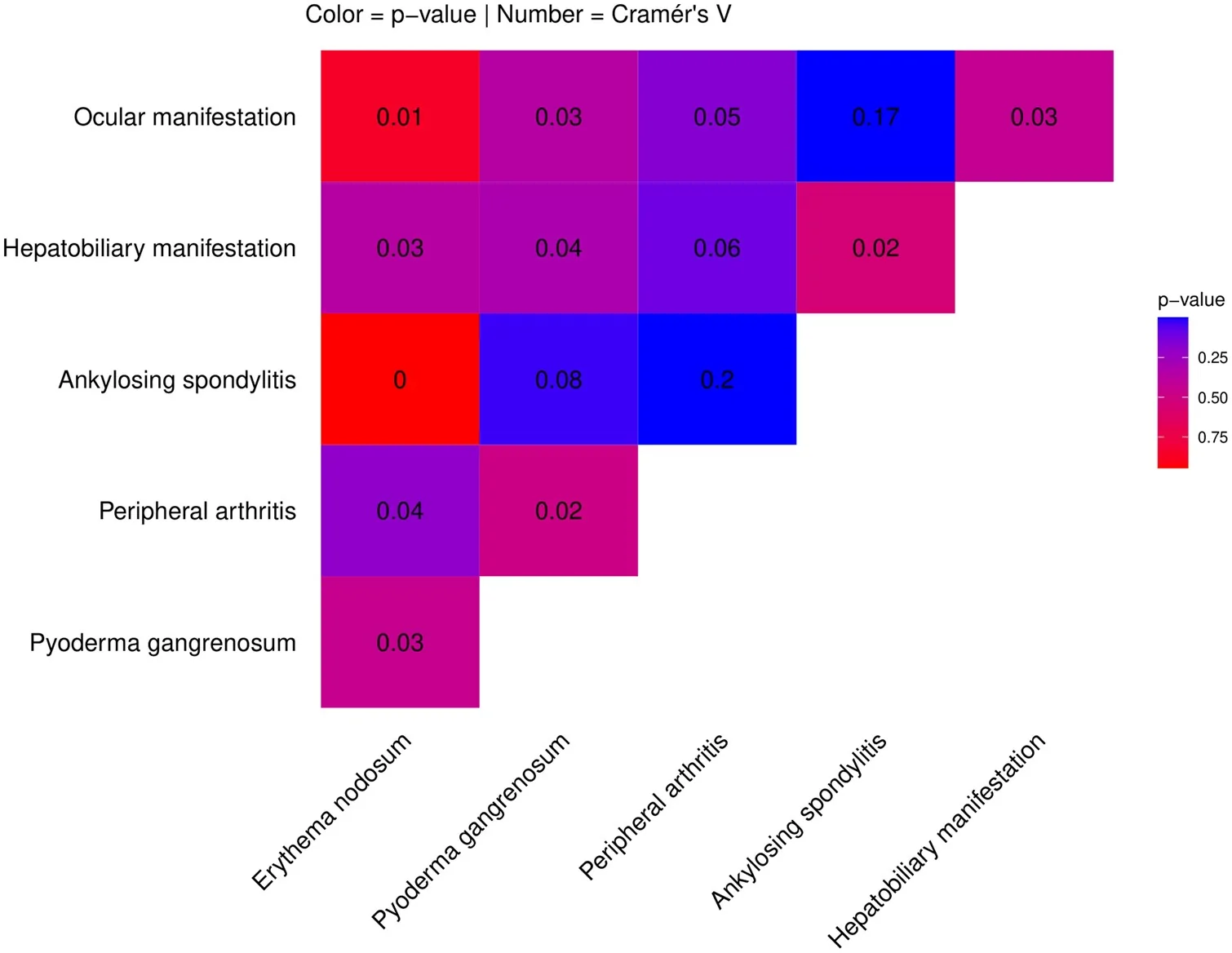
Interaction analysis of extraintestinal manifestations. Correlation matrix of various EIMs. Pairwise associations between EIMs are illustrated using Cramer’s V statistic, which quantifies the strength of association between categorical variables. The numbers within each box represent the estimated Cramer’s V value for each pair of EIMs. Higher Cramer’s V values indicate stronger associations. The color intensity corresponds to the chi-squared *P*-value for each association.

In the multivariable analysis, independent factors including sex, age at IBD diagnosis, disease duration, IBD subtype (UC vs CD), smoking exposure (ever vs never), history of IBD-related surgery, and the presence of a concomitant immune-mediated disease were evaluated for their association with the presence of EIMs. The results are presented in Figure 2 and Table S2. After adjustment for demographic and clinical covariates, female sex remained the strongest independent factor associated with EIM development (OR 2.88; 95% CI 2.08-4.02, *P* < .001). Longer IBD duration was also independently associated with an increased likelihood of EIMs, with each additional year of disease duration increasing the odds by approximately 2% (OR 1.02 per year; 95% CI 1.01-1.04, *P* = .002). Patients with CD had a significantly higher probability of developing EIMs than those with UC (OR 1.57; 95% CI 1.08-2.29, *P* = .018). Smoking exposure (OR 1.33; 95% CI 0.89-1.97, *P* = .15), concomitant immune-mediated disease (OR 1.56; 95% CI 0.91-2.66, *P* = .10), age at IBD diagnosis (OR 1.00; 95% CI 0.99-1.02, *P* = .549), and IBD-related surgery (OR 0.91; 95% CI 0.62-1.34, *P* = .66) were not independently associated with the presence of EIMs.

**Figure 2:**
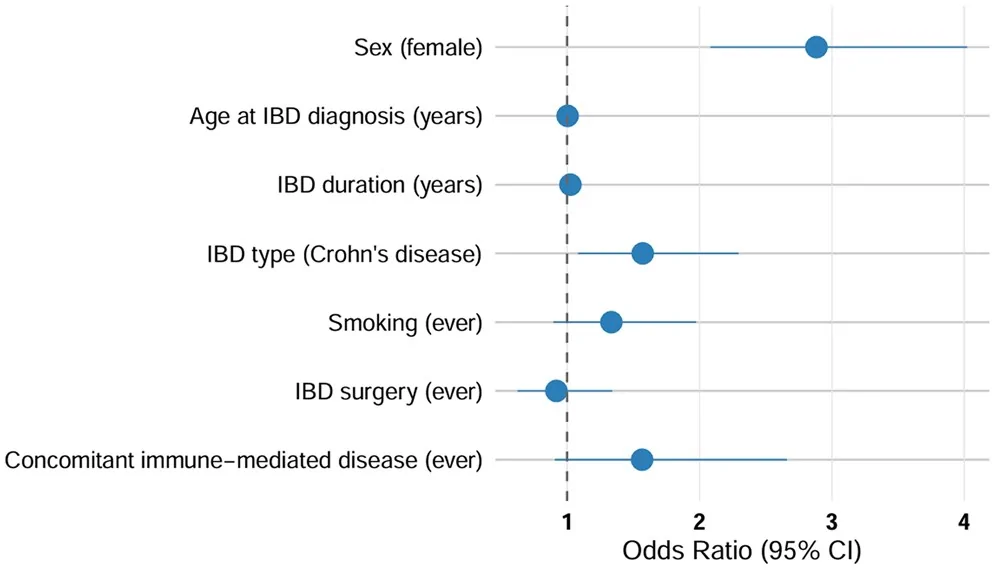
Multivariable analysis of factors associated with extraintestinal manifestations. The forest plot shows the odds ratios (ORs) and 95% confidence intervals derived from a multivariable logistic regression model that assessed factors independently associated with the presence of extraintestinal manifestations. The model included the following variables: sex, age at diagnosis of IBD, IBD duration, IBD type, smoking status (ever), history of IBD-related surgery and concomitant immune-mediated disease.

As a sensitivity analysis, a multivariable Cox proportional hazards regression accounting for variable follow-up duration was additionally performed (Table S3; Figure S2). The results were largely consistent with those of the multivariable logistic regression. Female sex remained independently associated with an increased risk of developing a first EIM (HR 2.84, 95% CI 2.06-3.92, *P* < .001). CD (HR 1.42, 95% CI 0.99-2.03, *P* = .055) and concomitant immune-mediated disease (HR 1.59, 95% CI 1.00-2.52, *P* = .051) showed borderline associations, whereas smoking exposure and previous IBD-related surgery were not significantly associated with time to first EIM.

### Timing of extraintestinal manifestations

A total of 227 patients developed at least one EIM. The timing of the first EIM relative to IBD diagnosis was analyzed to assess when EIMs may initially develop in patients with IBD. The median time to first EIM diagnosis was 60 months (IQR 5.5-156 months). The earliest recorded first EIM was diagnosed 144 months before IBD diagnosis, whereas the latest first EIM occurred 432 months after IBD diagnosis. Overall, 21 patients (9.3%) developed their first EIM concurrently with IBD diagnosis, while 26 patients (11.5%) developed their first EIM before IBD diagnosis. The majority of EIMs occurred after IBD diagnosis (Figure 3). In addition to the timing of the first EIM, the temporal distribution of all EIMs was assessed. A total of 306 EIMs were recorded. Twenty-five (8.2%) occurred concurrently with IBD diagnosis, while 33 (10.8%) preceded the diagnosis. The median timing of all EIMs was 72 months after IBD diagnosis (IQR 8-168), ranging from 144 months before to 432 months after diagnosis. Most EIMs developed after the diagnosis of IBD (Figure S1).

**Figure 3:**
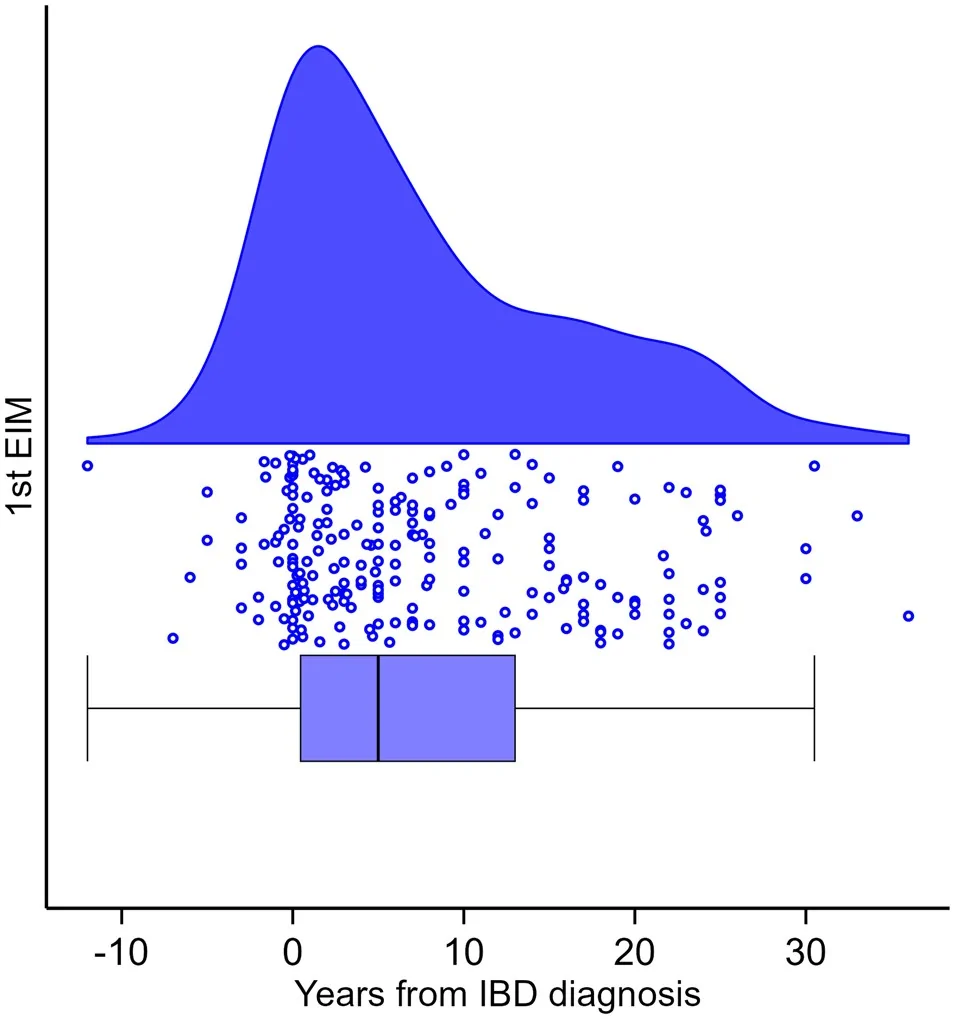
Timing of occurrence of the first extraintestinal manifestation relative to the diagnosis of inflammatory bowel disease.

Kaplan–Meier analysis demonstrated a continuous and time-dependent increase in the cumulative probability of developing EIMs over the disease course (Figure 4). Approximately 15% of patients developed an EIM within 5 years after IBD diagnosis, increasing to 27% after 15 years. The cumulative probability approached 50% after approximately 35 years of follow-up; however, estimates in the late follow-up period should be interpreted with caution because of the limited number of patients remaining at risk.

**Figure 4:**
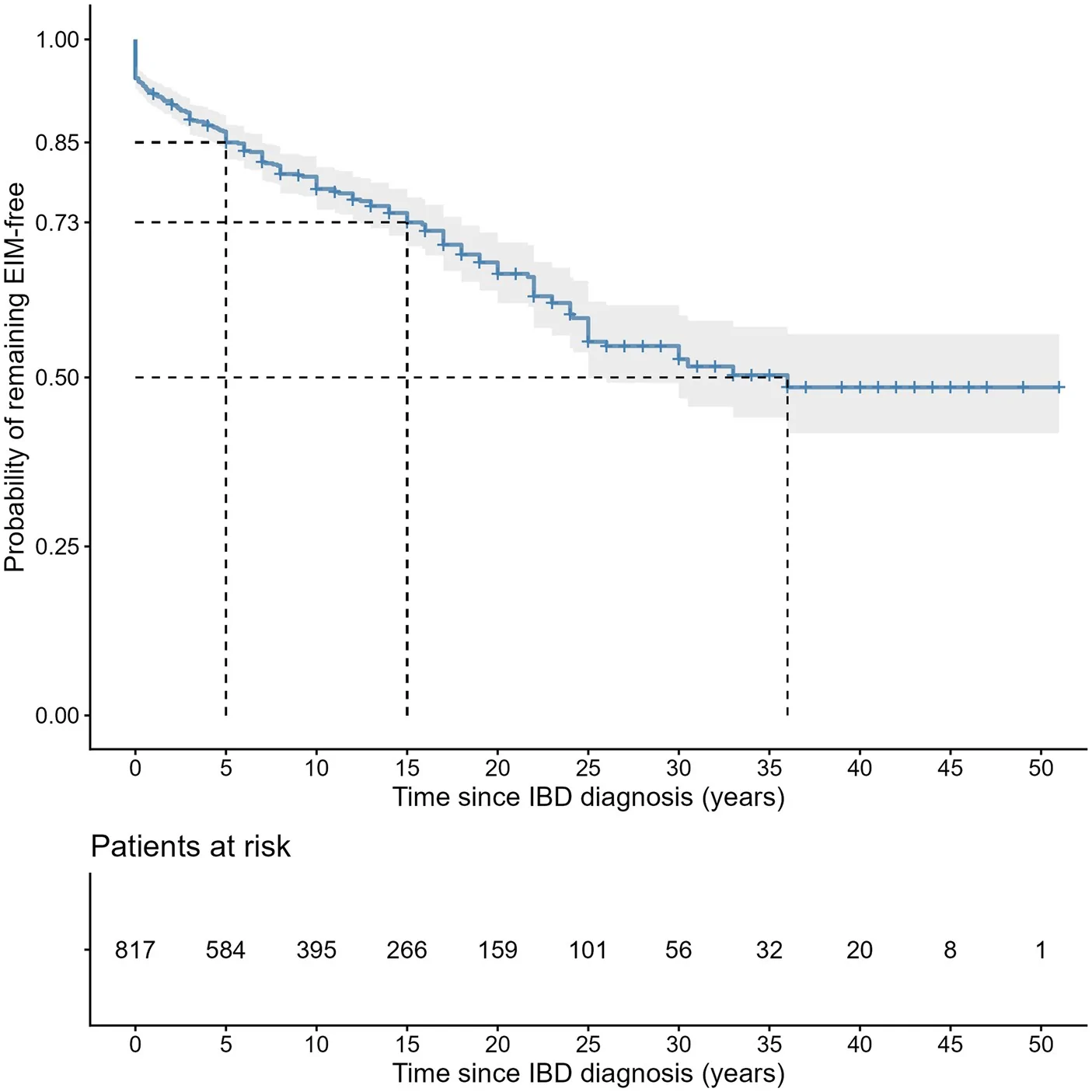
Kaplan–Meier curve illustrating the probability of remaining free from extraintestinal manifestations (EIMs) over time following the diagnosis of inflammatory bowel disease (IBD). Dashed vertical lines indicate selected time points at 5 and 15 years after IBD diagnosis, while the dashed horizontal line marks the 50% probability threshold. Shaded areas represent 95% confidence intervals. Numbers of patients at risk at each time point are shown below the x-axis.

## Discussion

In this large retrospective cohort of patients with IBD, EIMs were identified in more than one quarter of patients. Rheumatologic and cutaneous manifestations represented the most frequent EIM categories, while a proportion of patients experienced multiple EIMs during the course of their disease.

Rheumatologic manifestations were the most frequently identified EIMs in this study, in line with previous reports. In our cohort, rheumatologic manifestations accounted for 58.1% of all recorded EIMs, which is consistent with the existing literature, where rheumatologic manifestations are reported as the most common EIM category, often representing more than half of all EIMs and reaching proportions of up to 73% in some cohorts.^3^^,^^14^ Peripheral arthritis was the most common rheumatologic EIM (57.3% of rheumatic manifestations), as reported in other published studies.^11^^,^^15^ In the present study, only cases of arthritis confirmed by specialist evaluation were recorded, whereas arthralgia without objective evidence of arthritis was not included. Some studies have demonstrated that the prevalence of peripheral joint manifestations may be as high as 48% among patients with IBD; however, these estimates included arthralgias within the EIM category.^16^

Hepatobiliary manifestations were identified in 2.8% of patients overall. Among hepatobiliary manifestations, PSC was the most frequently observed, as expected, and was identified in 2.4% of patients overall, a finding that is consistent with previously published data.^17–19^ The reported prevalence of PSC among patients with IBD varies considerably across geographical regions. A recent meta-analysis found that PSC prevalence is highest in South America and lowest in Southeast Asia, with intermediate rates reported in Europe and North America.^20^ A large multinational Asian cohort demonstrated a lower prevalence of PSC in patients with IBD (0.92%) compared with the Western population.^21^ Consistent with previous observations, PSC occurred more frequently in patients with UC than in those with CD in our cohort (2.7% vs 2.2%). A higher odds of PSC in UC compared with CD (OR 1.69, 95% CI 1.24-2.29) has been demonstrated in meta-analyses, a pattern that appears to be consistent across both Western and Asian populations.^20–22^ Portal vein thrombosis accounted for the remaining hepatobiliary manifestations in our cohort (0.4%). Although uncommon, its inclusion within the hepatobiliary EIM category is consistent with a recent systematic review and meta-analysis including more than 1.7 million patients with IBD, which reported a pooled prevalence of 0.21% for portal vein thrombosis.^23^

Cutaneous manifestations have been reported in up to 15% of IBD patients at some point during the course of the disease.^24^^,^^25^ In our cohort, they affected 8.9% of the study population, with erythema nodosum and pyoderma gangrenosum being the most commonly observed conditions. Previous studies have demonstrated a higher risk of cutaneous EIMs among female patients. The odds ratio for cutaneous manifestations in females may range from 1.48 to 3.21, depending on the population and specific skin manifestation studied.^26^^,^^27^ An interesting finding of our study was the identification of cheilitis granulomatosa in four patients with CD, corresponding to 0.8% of the CD population. All diagnoses in our cohort were confirmed histologically by biopsy. Cheilitis granulomatosa represents a rare cutaneous EIM of IBD, for which data on true prevalence remain limited and which may be overlooked in routine clinical practice.^28^^,^^29^

Ocular manifestations constitute a clinically relevant, though less frequent, category of EIMs in IBD. Despite their lower prevalence compared with musculoskeletal and cutaneous manifestations, ocular involvement is of particular importance due to the risk of visual impairment. The most commonly reported ocular EIMs include episcleritis, scleritis, and uveitis.^30^ Across published cohorts, the prevalence of ocular manifestations in patients with IBD ranges from approximately 2% to 10%, depending on the population and study design.^31^^,^^32^

Female sex, disease duration and CD were found to be significantly associated with the development of EIMs, in accordance with previous studies.^33^ In the largest study to date, the SEXEII study from the ENEIDA registry (*n* = 51,595), female sex was identified as an independent risk factor for EIMs in both CD and UC (OR 1.48 in CD and UC).^27^ Another multicohort analysis including 12,083 patients corroborated that EIMs are more frequent in female patients and in those with CD.^4^ Potential mechanisms underlying this association include sex hormone–mediated modulation of immune responses, as estrogen and progesterone enhance humoral and cell-mediated immunity and may increase susceptibility to autoimmune phenomena. In addition, sex-linked genetic factors and differential expression of immune-related genes may further contribute to this risk.^34^^,^^35^ Mechanistically, CD is characterized by a dominant Th1/Th17 immune response, with increased production of interferon-γ, interleukin-17, and interleukin-22, as well as impaired regulatory T-cell activity. This leads to excessive activation of proinflammatory cytokines, notably tumor necrosis factor (TNF), interleukin-6 (IL-6), and interleukin-23, which drive both intestinal and systemic inflammation. Compared with UC, CD likely involves a broader dysregulation of mucosal and systemic immunity, attributable to its transmural pattern of inflammation.^36^

Although treatment exposure was comprehensively recorded to characterize the study cohort, it was not evaluated as a predictor of EIM occurrence because treatment selection in routine clinical practice is strongly influenced by disease phenotype and the presence of EIMs, resulting in confounding by indication. Nevertheless, previous studies have suggested differences in the risk of new-onset EIMs between gut-selective and systemic biologics,^37^^,^^38^ an area that warrants prospective investigation.

An additional noteworthy finding of our study is the observed trend toward an association between the development of EIMs and the presence of other immune-mediated disorders, such as Hashimoto thyroiditis and alopecia areata, which are not traditionally classified as classical EIMs of IBD. Although this association did not reach statistical significance in the multivariable analysis, it suggests that a broader immune-mediated disease burden may predispose patients to EIM development. From a clinical perspective, this finding underscores the need for increased vigilance by clinicians and highlights the importance of systematically considering a patient’s immune-mediated disease history in patients with IBD.

Furthermore, in nearly one fifth of patients who developed EIMs, EIM onset preceded or coincided with the diagnosis of IBD. Specifically, 20.7% of patients with EIMs experienced their first EIM before or concurrently with IBD diagnosis, underscoring that EIMs may represent an early feature of the disease rather than a late complication. This finding highlights the importance of maintaining a high index of suspicion for underlying IBD in patients presenting with immune-mediated extraintestinal symptoms, particularly when accompanied by gastrointestinal complaints. Previous cohort studies have similarly highlighted the temporal variability of EIM onset in relation to IBD diagnosis. Data from the Swiss IBD Cohort Study demonstrated that approximately 26% of patients developed their first EIM prior to the diagnosis of IBD, whereas 74% developed EIMs after IBD diagnosis.^12^ Likewise, a study by Zippi et al. including 811 Italian patients reported that 11% of EIMs occurred before IBD diagnosis, 19% concomitantly, and 70% after the diagnosis.^14^ The proportion of patients who developed an EIM prior to the diagnosis of IBD in our cohort was somewhat lower than that reported in previous studies. This difference may, at least in part, be attributable to the strict definition of EIMs applied in our analysis, which required specialist confirmation and relied on the documented date of diagnosis rather than the reported onset of symptoms. In addition, the retrospective design of our study, in contrast to the prospective Swiss IBD Cohort Study, may have limited the capture of EIMs occurring before IBD diagnosis. Furthermore, increased awareness of IBD among clinicians in contemporary clinical practice, compared with earlier periods, may have facilitated earlier recognition and diagnosis of IBD, thereby reducing the time window during which EIMs could precede overt intestinal disease. Despite these differences, a relevant proportion of patients developed an EIM before or at the time of IBD diagnosis, highlighting the clinical importance of extraintestinal symptoms in the diagnostic evaluation of patients with suspected IBD.

This study has several limitations that should be acknowledged. First, the retrospective design is inherently associated with potential biases. Secondly, EIMs were recorded only when the diagnosis was confirmed by the appropriate specialist. While this enhances diagnostic accuracy and internal validity, it may have led to an underestimation of the true prevalence of EIMs. Mild, transient, or subclinical manifestations, as well as symptoms that did not prompt specialist referral, were likely underrepresented. In addition, arthralgia without objective evidence of arthritis was not included, in contrast to some previous studies, which may limit direct comparability across cohorts. Thirdly, the timing of EIMs was determined based on the date of formal diagnosis by the relevant specialist, rather than the reported onset of symptoms. This approach may have affected the evaluation of the temporal relationship between EIMs and IBD diagnosis, especially for symptoms that appeared before intestinal disease was recognized. Moreover, although only specialist-confirmed manifestations considered related to the underlying IBD were included, the retrospective design does not allow definitive confirmation that manifestations diagnosed years before intestinal disease onset were causally related to IBD rather than representing independent conditions that preceded IBD by chance. This limitation should be considered when interpreting the proportion of EIMs occurring before IBD diagnosis. Furthermore, the classification of concurrent EIM and IBD diagnoses using a ±2-week interval was based on a pragmatic definition. Delays in referral to the appropriate specialist or scheduling of diagnostic assessments may have influenced the recorded timing of EIM diagnosis in some patients, potentially resulting in misclassification around this time window. Fourth, this was a single-center study conducted at a tertiary referral university hospital, which may limit the generalizability of the results. Patients followed at such centers often have more severe or complex disease phenotypes, as reflected by the high rates of biologic therapy exposure and IBD-related surgery in this cohort. Finally, although multivariable analyses were performed to identify factors independently associated with EIM development, residual confounding by unmeasured or unavailable variables cannot be excluded, including intestinal disease activity. Furthermore, treatment status at the time of EIM diagnosis, including the specific advanced therapy being administered, was not systematically available in the dataset and therefore could not be analyzed. Notably, objective data on intestinal disease activity at the time of EIM diagnosis were not included in the dataset due to substantial heterogeneity in data availability; while some patients had recent endoscopic assessments, others had only biochemical markers available, and in a subset of patients, disease activity was documented solely based on clinical evaluation.

Despite these limitations, this study has several important strengths. These include the large, well-characterized cohort, the long duration of follow-up and the assessment of a broad spectrum of EIMs. The strict requirement for specialist-confirmed EIM diagnoses enhances diagnostic reliability, while the detailed analysis of timing and associated risk factors provides clinically relevant insights into the natural history of EIMs in patients with IBD.

In conclusion, EIMs were observed in more than one quarter of patients with IBD, with rheumatologic and cutaneous manifestations being the most common. Female sex, disease duration and CD were independently associated with a higher risk of EIM development. Notably, a considerable proportion of EIMs occurred before or concurrently with IBD diagnosis and their incidence increased steadily over time, underscoring the importance of early recognition and long-term surveillance of extraintestinal disease.

## Supplementary Material

otag100_Supplementary_Data

## Data Availability

The data supporting the findings of this study are available from the corresponding author upon reasonable request.
