# Supplementary material for: Prevalence, timing and risk factors of extraintestinal manifestations in inflammatory bowel disease: a retrospective cohort study from a German tertiary referral center
Source: Crohns Colitis 360. 2026 Sep 2;8(4):otag100. doi: 10.1093/crocol/otag100 (PMC13637596; doi:10.1093/crocol/otag100)
Supplement: Supplementary file 1 [file otag100_supplementary_data.zip › Supplementary Material..docx]

**Supplementary Table S1**. Pairwise associations between extraintestinal manifestations. Values represent Cramer's V statistics derived from pairwise chi-squared tests, indicating the strength of association between pairs of extraintestinal manifestations. Higher values indicate stronger associations. Cramer's V values of approximately 0.10, 0.30, and 0.50 were interpreted as weak, moderate, and strong associations, respectively.

| **EIMs (row × column)** | **Erythema nodosum** | **Pyoderma gangrenosum** | **Peripheral arthritis** | **Ankylosing spondylitis** | **Hepatobiliary manifestations** | **Ocular manifestations** |
| --- | --- | --- | --- | --- | --- | --- |
| **Erythema nodosum** | 1 | 0.0267 | 0.04439 | 0.002415 | 0.03171 | 0.006116 |
| **Pyoderma gangrenosum** | 0.0267 | 1 | 0.02407 | 0.07639 | 0.0354 | 0.03184 |
| **Peripheral arthritis** | 0.04439 | 0.02407 | 1 | 0.2049 | 0.05565 | 0.04765 |
| **Ankylosing spondylitis** | 0.002415 | 0.07639 | 0.2049 | 1 | 0.02102 | 0.1678 |
| **Hepatobiliary manifestations** | 0.03171 | 0.0354 | 0.05565 | 0.02102 | 1 | 0.02764 |
| **Ocular manifestations** | 0.006116 | 0.03184 | 0.04765 | 0.1678 | 0.02764 | 1 |

**Supplementary Table S2**. Multivariable logistic regression analysis for factors associated with extraintestinal manifestations.

| **Variable** | **Odds Ratio (OR)** | **95% CI** | **p-value** |
| --- | --- | --- | --- |
| **Female** | 2.88 | 2.08–4.02 | <0.001 |
| **Age at diagnosis of IBD** | 1.00 | 0.99–1.02 | 0.549 |
| **IBD Duration (years)** | 1.02 | 1.01-1.04 | 0.002 |
| **Crohn’s disease (vs Ulcerative colitis)** | 1.57 | 1.08-2.29 | 0.018 |
| **Smoking (ever)** | 1.33 | 0.89–1.97 | 0.15 |
| **IBD-related surgery** | 0.91 | 0.62–1.34 | 0.66 |
| **Concomitant immune-mediated disease** | 1.56 | 0.91–2.66 | 0.10 |

**Supplementary Figure S1.** Timing of occurrence of all extraintestinal manifestations relative to the diagnosis of inflammatory bowel disease.

| **Variable** | **HR (95% CI)** | **p-value** |
| --- | --- | --- |
| **Female** | 2.84 (2.06-3.92 | <0.001 |
| **Age at diagnosis of IBD** | 1.01 (1.00-1.003) | 0.024 |
| **Crohn’s disease (vs Ulcerative colitis)** | 1.42 (0.99-2.03) | 0.055 |
| **Smoking exposure (ever vs never)** | 1.08 (0.76-1.53) | 0.668 |
| **IBD-related surgery** | 0.74 (0.54-1.03) | 0.079 |
| **Concomitant immune-mediated disease** | 1.59 (1.00-2.52) | 0.051 |

**Supplementary Table S3**. Multivariable Cox regression analysis for factors associated with extraintestinal manifestations.

*Hazard ratios (HRs) were estimated using a multivariable Cox proportional hazards model. Time at risk was calculated from the date of IBD diagnosis to the first extraintestinal manifestation or last follow-up. Patients whose first EIM occurred before IBD diagnosis (n = 26) were excluded because the event preceded the defined time origin.*

**Supplementary Figure S2.** Multivariable Cox proportional hazards regression analysis of factors associated with extraintestinal manifestations in inflammatory bowel disease.
